# Patchwork sequencing of tomato San Marzano and Vesuviano varieties highlights genome-wide variations

**DOI:** 10.1186/1471-2164-15-138

**Published:** 2014-02-18

**Authors:** Maria Raffaella Ercolano, Adriana Sacco, Francesca Ferriello, Raffaella D’Alessandro, Paola Tononi, Alessandra Traini, Amalia Barone, Elisa Zago, Maria Luisa Chiusano, Genny Buson, Massimo Delledonne, Luigi Frusciante

**Affiliations:** 1 Department of Agriculture Sciences, University of Naples Federico II, Via Universita’ 100, 80055 Portici, Italy; 2 Dipartimento di Biotecnologie, Università degli Studi di Verona, Strada le Grazie, 15, 37134 Verona, Italy; 3 Present address: East Malling Research, New Road, East Malling, Kent ME19 6BJ, UK

**Keywords:** Combined assembling, Fruit quality, NGS sequencing, SNPs, *Solanum lycopersicum*

## Abstract

**Background:**

Investigation of tomato genetic resources is a crucial issue for better straight evolution and genetic studies as well as tomato breeding strategies. Traditional Vesuviano and San Marzano varieties grown in Campania region (Southern Italy) are famous for their remarkable fruit quality. Owing to their economic and social importance is crucial to understand the genetic basis of their unique traits.

**Results:**

Here, we present the draft genome sequences of tomato Vesuviano and San Marzano genome. A 40x genome coverage was obtained from a hybrid Illumina paired-end reads assembling that combines *de novo* assembly with iterative mapping to the reference *S. lycopersicum* genome (SL2.40). Insertions, deletions and SNP variants were carefully measured. When assessed on the basis of the reference annotation, 30% of protein-coding genes are predicted to have variants in both varieties. Copy genes number and gene location were assessed by mRNA transcripts mapping, showing a closer relationship of San Marzano with reference genome. Distinctive variations in key genes and transcription/regulation factors related to fruit quality have been revealed for both cultivars.

**Conclusions:**

The effort performed highlighted varieties relationships and important variants in fruit key processes useful to dissect the path from sequence variant to phenotype.

## Background

Tomato (*Solanum lycopersicum*) is one of the most economically important vegetable crops worldwide. It is a rich source of micronutrients for human diet and a model species for fruit quality. Investigation of tomato genetic resources is a crucial issue for better straight evolution and genetic studies as well as tomato breeding strategies.

Since the late 18th and throughout the 19th and early 20th centuries a huge array of crosses and selection activities has taken place in Europe giving rise to a rich collection of tomato landraces [1,2]. In particular, an extensive selection work was performed in Italy by “Campania” farmers that developed several varieties adapted to local conditions and with quality requirements well delineated for specific uses. Among them, Vesuviano (RSV) and San Marzano (SM) varieties, grown in rich volcanic soil surrounding Vesuvius, are considered important models for fruit quality parameters. The Vesuviano has been cultivated on the Vesuvio hill, since the end of 19th century. It was selected by the local farmers because of its tolerance to the drought [3]. The origin of the San Marzano variety is very debatable. Some people report that San Marzano was a mutant from the local varieties (Corbarino); other people report that San Marzano was a natural hybridization between the grown varieties in the Agro-Sarnese-Nocerino area. Certainly, the cultivation of the San Marzano ecotype started in the years 1903–1904 in the Agro-Sarnese-Nocerino area becoming immediately a top variety for peeling [4]. Previous studies revealed that presently cultivated Vesuvio and San Marzano genotypes revealed peculiar sensory profiles in perception of sweetness and sourness [5,6]. San Marzano and Vesuvio fruits can purchased by at a price that is nearly five times higher than that of other varieties [7].

The advent of genomics era has brought a substantial increase in generation of data, knowledge and tools that can be employed in applied research. Candidate genes for important traits can be identified, and exploring functional nucleotide polymorphisms within genes of interest can facilitate breeders in combining favourable alleles. The decoding of the Heinz 1706 tomato reference genome SL2.40 will allow a better understanding of genetic basis of agronomic traits for developing novel genotypes [8,9]. Genome sequences and genomic tools offer exciting new perspectives and opportunities to track rates of sequence divergence over time, and provide hints about how genes evolve and generate new products by re-organization and shuffling of genomic sequences. Variant catalogues, however, will remain incomplete if forms of variation are undocumented. Good genome coverage is required to improve variant detection and accuracy and to study the polymorphism distribution across genomes. Genetic diversity studies have been improved by Next Generation Sequencing (NGS) based approaches [10,11]. However, interpreting the effect of genetic variation has typically relied on a reference genome. Indeed, alignment-consensus methods may have serious limitations in describing polymorphic regions and may also cause biases in interpreting the effect of variation on coding sequences. On the other hand *de novo* assembly approaches may theoretically overcome such problems, but pose a number of challenges due for example to repetitive sequences, low complexity sequences and closely related paralogs [12]. Alternative hybrid approaches can overcome limitations of alignment-consensus methods [13,14], allowing to capture a broader spectrum of sequence variation comparing genome with or without reference genome [15].

Here we describe the generation and analysis of San Marzano and Vesuviano tomato genome sequences. First, we reconstructed the genomes using a combination of iterative mapping and de novo assembly. Then, we annotated genes and documented the variation discovered, describing the typology and the distribution of variants between genotypes at chromosome level. Finally, as proof of concept we assessed the variability in fruit quality related genes, exploring the quantitative and qualitative impact of functional variants. Data produced can be helpful to investigate the genomic origins of phenotypic variation as well as to perform breeding programs.

## Methods

### Sequencing

A total amount of 2.5 μg of genomic DNA was sonicated with Covaris S2 instrument to obtain 400 bp fragments. DNA library preparation of SM and RSV tomato varieties (Additional file 1: Figure S1) was carried out using the TruSeq DNA Sample Prep Kit v2 (Illumina, San Diego, CA) accordingly to manufacturer instructions. RNA library preparation of SM and RSV tomato berry samples was carried out using the TruSeq RNA Sample Prep Kit v2 (Illumina, San Diego, CA) accordingly to manufacturer instructions.

Quality control of libraries was performed using High Sensitivity DNA Kit (Agilent, Wokingham, UK) and an accurate quantification was made using qPCR with KAPA Library Quantification kit (KapaBiosystems, USA). Libraries were then pooled and sequenced using Illumina Hiseq 1000 and applying standard Illumina protocols with TruSeq SBS Kit v3-HS and TruSeq PE Cluster Kit v3-cBot-HS kits (lllumina, USA). Libraries were sequenced with an Illumina Hiseq 1000 sequencer (Illumina Inc., San Diego, CA, USA) and 100-bp paired-end sequences were generated.

### Genome assembly and annotation transfer

Genome reconstruction and variants identification were performed with the IMR/DENOM ver. 0.3.3 pipeline [14] using default parameters and the SL2.40 tomato genome [8] as reference. Repeats annotation was performed with RepeatMasker (v. open-3.3.0) using a custom redundant database available from SolGenomics website (ftp://ftp.solgenomics.net/tomato_genome/repeats/). ITAG 2.3 gene annotation was translated to the tomato reconstructed genomes by taking into account variants identified by IMR/DENOM pipeline and adjusting the coordinates accordingly using a custom software (http://ddlab.sci.univr.it/downloads/translate_coordinates.exe).

### Mapping of transcript sequences

We independently mapped the 34,727 coding sequences (CDSs) [16,17] defined by the *Solanum lycopersicum* genome annotation to identify similarities versus RSV and SM tomato genomes using GenomeThreader [18], CDSs were also re-mapped versus SL2.40 to compare results between the three different genotypes. We filtered out alignments at similarity thresholds lower than 80% coverage and 90% identity. Correspondence among the loci in the three genotypes was defined on the basis of conserved loci position analyses at chromosome level and their distribution is reported using the CIRCOS program [19].

### Variants analysis and validation

Identified variants between SL2.40 genome SM and RSV genotype were analysed using SnpEff version 2.1b (build 2012-04-20) [20] to predict their the effect on the genes in ITAG2.3 annotation. CDS non-synonymous variants were also submitted to PROVEAN (Protein Variation Effect Analyzer algorithm) analysis, which predicts the functional impact for all classes of protein sequence variations such as single amino acid substitutions but also insertions, deletions, and multiple substitutions [21]. To validate the identified SNPs, paired-end RNA-Seq reads (100 bp) from SM and RSV fruit samples were mapped against the reference genome SL2.40. SNPs were called using SAMtools 0.1.18 [22] with a minimum read depth threshold of 6 and then compared with genomic reads using BEDTools 2.17.0 software [23].

### Enrichment analysis

Our attention was focused on non-synonymous SNPs located in CDS belonging to four gene classes related to fruit quality (ascorbate biosynthesis; MEP/carotenoid pathway; ethylene-related genes; cell wall related genes); transcription factors and transcription regulators potentially involved in fruit ripening process. To evaluate if significant enrichment was present in specific metabolic pathways, an enrichment analysis based on Gene Ontology (GO) terms classification [24] was performed. We associated a GO term to each gene containing a non-synonymous coding variation running the BLAST2GO platform [25]. The data sets obtained were compared to the entire set of tomato genes with GO annotation (SOL Genomics. http://solgenomics.net/).

We performed a singular enrichment analysis (SEA) [26] which allows testing annotation terms against a list of interesting genes [27].

We used a hypergeometric test to compare each class to the reference background of genes. Hochberg (FDR) statistical correction was applied and a significance level of 0.05 was set. The minimum number of mapping entries was set as 1 to observe any significant enrichment. Only gene classes with a least 20 protein members (transcription factors, transcription regulators and cell wall) were subject to enrichment analysis.

#### Data access

All next-generation sequencing data are available in the Sequence Reads Archive (SRA) [SRA:SRP027562] Variants data in Snps, Deletions and Insertions (SDI) file format are available on SOL Genomics Network (SGN) website (ftp://ftp.solgenomics.net).

## Results

### Genome assembly

We sequenced Vesuviano (RSV) and San Marzano (SM) tomato varieties using Illumina 100 bp paired-end reads with an insert size of about 250 bp. We obtained 155,751,012 (X2) pareid-end reads for RSV and 177,758,218 (X2) paired-end reads for SM that, considering an expected size of about 900 Mb [8], correspond to an average expected depth of about 34.6x and 39.5x genome equivalent, respectively (Additional file 2: Table S1). We chose to use a genome reconstruction method based on a combination of iterative read mapping against the tomato reference genome and de novo assembly that is able to describe complex loci on a single pass alignment [13] (Additional file 3: Figure S2). A similar number of mobile elements (63%) and outstanding proportion of LTR elements (93% of occupied length) with SL2.40 genome was found (Additional file 2: Table S2).

The size of the assembled genomes is very similar (99.8%) to the reference genome (Additional file 2: Table S3). The slightly lower size observed in the reconstructed genomes may be related to a low efficiency of the method in detecting long insertions. We aligned the reads to the final assemblies to detect regions with a low read coverage, which may correspond to complex polymorphisms. The average N50 length of contiguous regions between polymorphic regions was of 77.5 Kbp and 72.7 Kbp for RSV and SM, respectively, while polymorphic regions sizes had a maximum of 88.7 Kbp in RSV and with an average size of 1.4 Kbp in both varieties (Additional file 2: Table S4). These polymorphic regions insist on 368 genes for the RSV and 328 genes for SM, and 283 of the genes interested by polymorphic regions are in common between the two varieties. Interestingly, SNPs distribution on regions of chromosome 9 and 11, implicated in the introgression of *S.pimpinellifolium* disease resistance loci into “SL2.40”, showed higher density than average (Additional file 4: Figure S3), confirming previous findings. [8]. We detected 206,867 and 177,179 single base variants compared to the reference genome for RSV and SM respectively, of these 3,343 were shared between the two genotypes (Table 1). A small fraction (3.3% in average) of the single base variants was ambiguous and, most probably, corresponded to heterozygous variants or misalignments due to repeated sequences. In fact, 61% (RSV) and 63% (SM) of the putative heterozygous variants in either cultivar were located in annotated repeats and are most probably an artefact. We also identified a fairly large number of indels or unbalanced insertions (258,023 in global considering both varieties). Most SNPs were detected in intergenic and intronic regions (Table 2); as a whole the SNPs affected 23,220 and 20,353 genes for RSV and SM, respectively. Comparison with RNA-Seq reads from RSV and SM samples showed that approximately 90% of the SNPs covered with a minimum read depth of 6 were validated (data not shown).

**Table 1:** Variants statistics

	**RSV**	**SM**	**Common**
SNPs	206,867 (199,502)	177,179 (169,704)	3,343 (3,160)
Deletions	46,433	44,561	25,208
Insertions	77,882	75,984	55,668
Unbalanced insertions	6,537	6,626	1,343
**Total**	**337,719**	**304,350**	**85,562**

Number of sequence variants in Vesuviano (RSV) and San Marzano (SM) varieties compared to the reference genome (SL240) In brackets is reported the number of unambiguous SNPs In the last column the number of variants in common between the two varieties is shown.

**Table 2:** Counts of identified SNPs and of genes affected by them

	**RSV**		**SM**	
	**SNPs**	**Genes**	**SNPs**	**Genes**
Intergenic	188,769 (181,758)	16,705 (16,247)	158,964 (151,971)	14,530 (13,939)
Intronic	11,907 (11,766)	2,880 (2,836)	12,232 (11,979)	2,576 (2,490)
UTR	624 (610)	381 (376)	686 (671)	361 (353)
CDS	5,567 (5,368)	3,254 (3,132)	5,297 (5,083)	2,886 (2,752)
**Total**	**206,867 (199,502)**	**23,220 (22,591)**	**177,179 (169,704)**	**20,353 (19,534)**

Results are given distinguishing in which region the SNPs are found, if they are in intergenic regions, in genes, in exonic regions or in the coding regions of genes using the ITAG23 annotation of reference genome For variants that insists on a gene, a count of affected genes is also given In brackets are given the numbers of the unambiguous SNPs.

Indels sizes varied from single base up to 6,011 bp in the case of insertions and 36,162 bp in the case of deletions. While the majority of the indels were shorter than 6 bases, we detected 105 insertions longer than 100 bp in RSV affecting 62 genes and 97 in SM affecting 60 genes (Table 3). Moreover, we detected 2,499 deletions longer than 100 bp in RSV affecting 1,081 genes and 2,461 in SM affecting 1,042 genes (Table 4, Additional file 2: Table S4). However, we noticed that while SNPs were mostly specific of each cultivar, most of the insertions (71.4% of RSV insertions; 73.3% of SM insertions) and deletions (54.3% of RSV deletions; 56.6% of SM deletions) detected in each variety were shared with the other genotype (Table 1) and occurred with an average frequency of 1 indel every 6 Kb. These findings resemble quite closely the frequency of estimated indel error rates reported for the reference genome SL2.40 (1 every 6.4 Kbp) [8]; and suggest that common indels may be due to errors in the reference genome rather than to true indels.

**Table 3:** Insertion statistics

		**Insertions**			
	**Feature**	**RSV**		**SM**	
		**Variants**	**Genes**	**Variants**	**Genes**
Total	Intergenic	69,615	18,730	67,850	18,432
	Intronic	7,187	4,069	7,096	4,010
	UTR	557	485	567	489
	CDS	523	418	471	400
Length < 6	Intergenic	63,307	18,006	62,241	17,736
	Intronic	6,846	3,975	6,791	3,929
	UTR	535	466	553	478
	CDS	381	321	362	315
Length > 100	Intergenic	97	54	91	54
	Intronic	4	4	2	2
	UTR	0	0	0	0
	CDS	4	4	4	4

Statistics of the number of insertions and the number of corresponding annotated genes.

**Table 4:** Deletion statistics

		**Deletions**			
	**Feature**	**RSV**		**SM**	
		**Variants**	**Genes**	**Variants**	**Genes**
Total	Intergenic	43,385	9,909	41,604	9459
	Intronic	2326	1384	2317	1354
	UTR	141	110	147	115
	CDS	581	472	493	404
Length < 6	Intergenic	34,567	8103	32,974	7696
	Intronic	1898	1190	1914	1162
	UTR	114	88	114	87
	CDS	369	296	331	257
Length > 100	Intergenic	2365	950	2337	920
	Intronic	80	77	80	78
	UTR	6	6	7	7
	CDS	48	48	37	37

Statistics of the number of deletions and the number of corresponding annotated gene.

### Gene annotation

**Figure 1:**
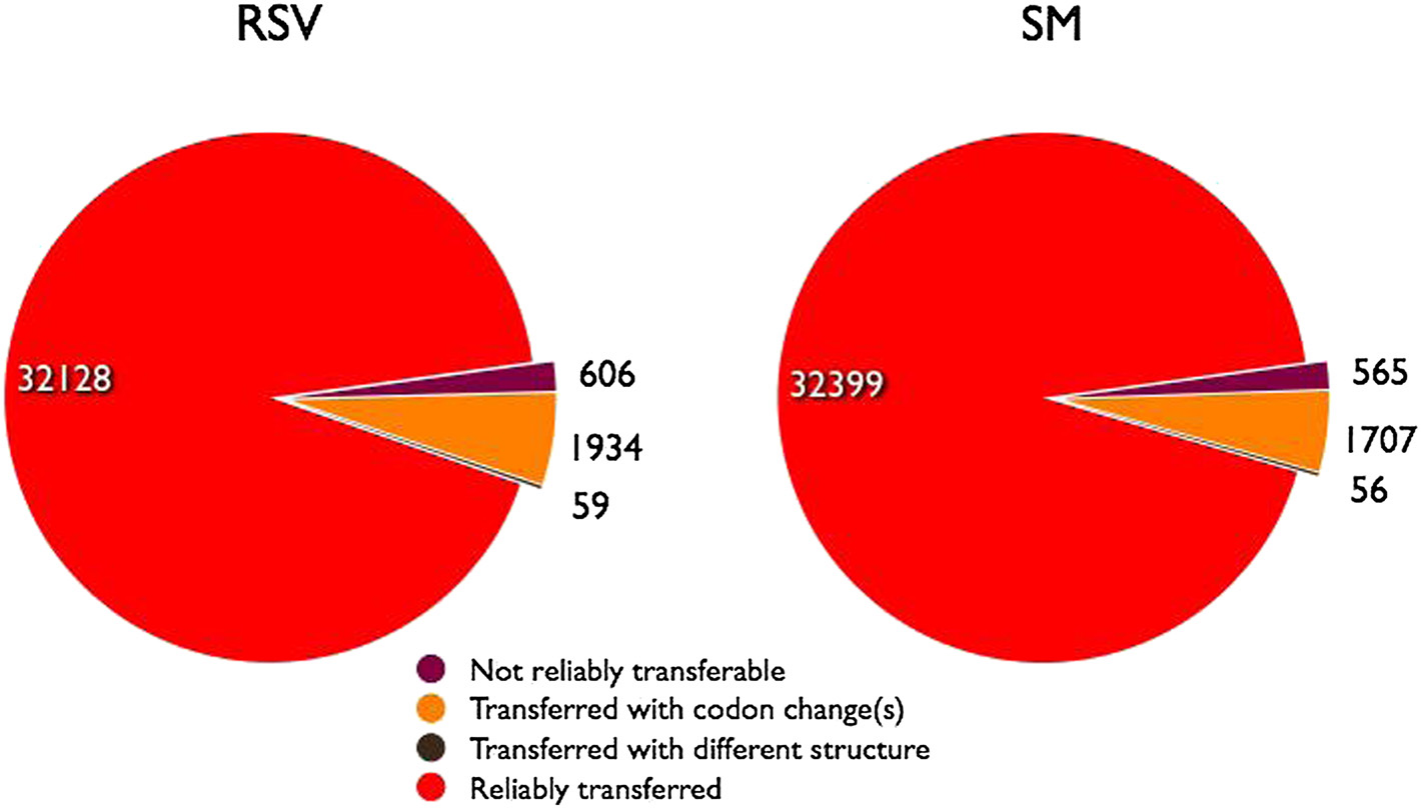
**Statistics of annotation translation.** Statistics of the ITAG23 annotation translated from the reference assembly to the Vesuviano (RSV) and San Marzano (SM) genomes.

We took advantage of the existing high quality *Solanum lycopersicum* reference annotation (ITAG2.3) released by the International Tomato Genome Sequencing Consortium [8] to annotate the assembled genomes for the RSV and SM varieties. The original annotations were transferred taking into account the cumulative effect of insertions and deletions along the whole length of the chromosomes. In order to evaluate the reliability of the transferred annotations we analysed the potential effect of detected variants projecting them on the corresponding protein coding sequences in each variety. Most variants were located outside the gene loci with only a smaller fraction harbouring SNPs or indels inside their coding sequences (Tables 2, 3 and 4). In particular, we found that most of the proteins encoded by RSV (98.1%) and SM genes (98.2%) were not affected by potentially disrupting mutations and were reliably transferred to the corresponding genomes (Figure 1). A small number of annotations (≈0.1%) was predicted to have an altered gene structure due to mutations in splice sites and were classified as “transferred with putative altered structure”. Moreover, 606 RSV and 565 SM genes, corresponding respectively to 1.7% and 1.6% of the total annotations, were predicted to be potentially affected by disrupting mutations such as frameshifts and alteration of the start or stop codon and could not be reliably transferred (Figure 1 and Additional file 2: Table S5).

### Transcript sequences mapping

**Figure 2:**
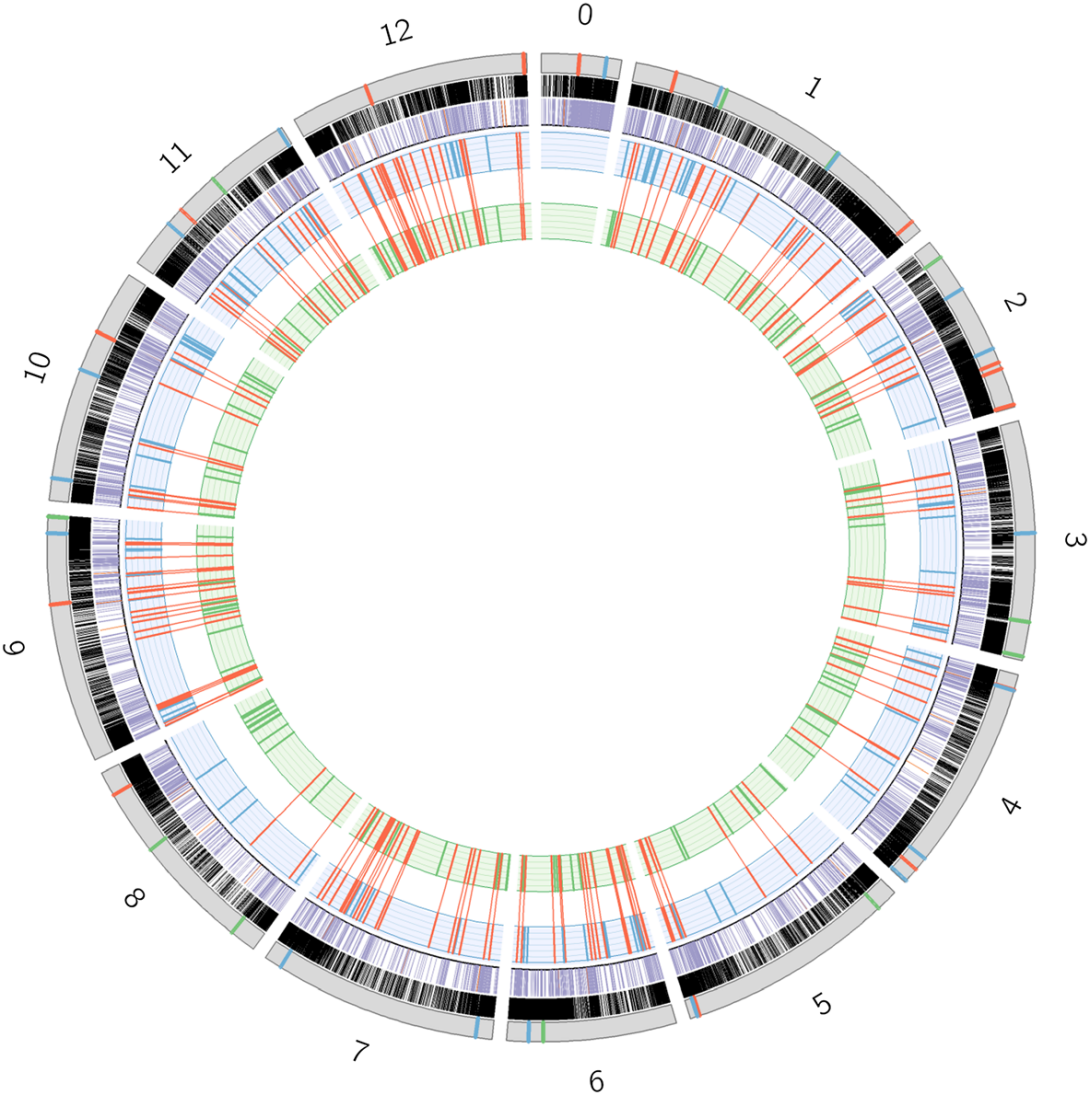
**Main results from ITAG23 gene mapping on SL240, Vesuviano (RSV) and San Marzano (SM) tomato genomes.** Grey bars represent the twelve tomato chromosomes and chromosome 0 The distribution of the genes within the SL240 genome is shown by black lines The distribution of genes mapped more than once is shown in the following circle with purple lines when the redundancy is equal or lower than 30 copies and with orange lines when genes re-mapped more than 30 times SM and RSV specific behaviours are indicated in green and in blue, respectively, in the whole picture, while the common behaviours among the two genotypes are indicated in red In particular, on each chromosome (grey bars) colored lines indicate the distribution of the S lycopersicum genes which are not mapped in the two SM and RSV genotypes The two inner circles indicate the distribution of genes that are specific to RSV (circle with a light blue background) and to SM (circle with a light green background) genotypes, along the respective pseudomolecules Common genes identified exclusively in chromosome positions from the two newly sequenced genotypes are in red and are linked by red connectors between the two inner circles to better highlight the conserved positions of these genes in the two newly sequenced genomes

**Figure 3:**
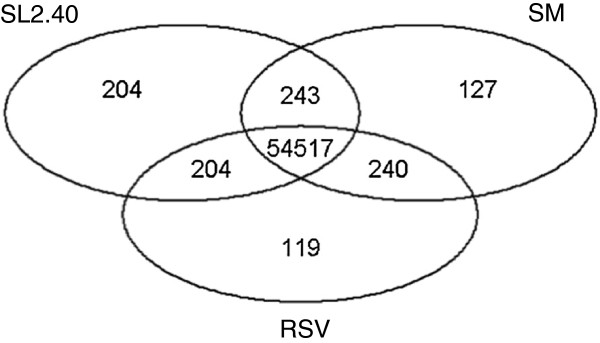
Venn diagram of loci that share the same relative position in the reference SL240,Vesuviano (RSV) and San Marzano (SM) genomes.

We also mapped the 34,727 CDSs defined by the *Solanum lycopersicum* genome *versus* RSV, SM and the SL2.40 reference genomes. As expected, since the analyses was conducted at coding sequence level, better highlighted similarities between loci coding for the same protein family, and also some CDSs mapped more than one time along the three different genotypes (Table 5). Figure 2 reported the distribution of these loci over the SL2.40 genome. The distribution of *S. lycopersicum* genes for which CDSs are not mapped in SM and RSV, are highlighted by coloured lines over the grey chromosome bars. The gene loci, resulting from the mapping procedure, were compared among the twelve chromosomes of each genotype. This permitted to define any difference at genome level that could be associated to an unsuccessful mapping of CDS sequences, pointing out variability of gene loci distribution, of their protein coding exon-intron organization, or at nucleotide level, detectable thanks to difference of similarity score between the genotypes. Figure 3 reported a Venn diagram indicating the number of loci that shared the same relative position in the three genomes, and those that are present only in two or even one of the genotypes. In SL2.40 and SM genomes 243 loci maintained the same position, while in SL2.40 and RSV only 204. Among the 54,517 loci that are in common between the three different genotypes, 3,411 in total showed different similarity scores (in terms of percentage of identity and coverage of the aligned mRNA *versus* the genome) when compared with the reference genome loci organization. Specifically, RSV showed differences in 2,224 loci, while SM showed difference in 1,610 loci. Moreover, when counting the number of loci with identical similarity score, 881 loci from SM are identical to SL2.40 ones, while 596 genes in RSV resulted identical to SL2.40. These evidences suggest a higher similarity between SL2.40 and SM genomes.

**Table 5:** **Number of tomato genes mapped *versus *** **the reference tomato (SL240), the Vesuviano (RSV) and San Marzano (SM) genomes**

	**SL240**	**RSV**	**SM**
1 match	29,844	29,940	29,934
>1 match	4,755	4,621	4,638
total	34,600	34,561	34,572

The number of genes resulting in one match or in multiple matches are indicated.

### Analysis of genetic variants in fruit quality related genes

**Figure 4:**
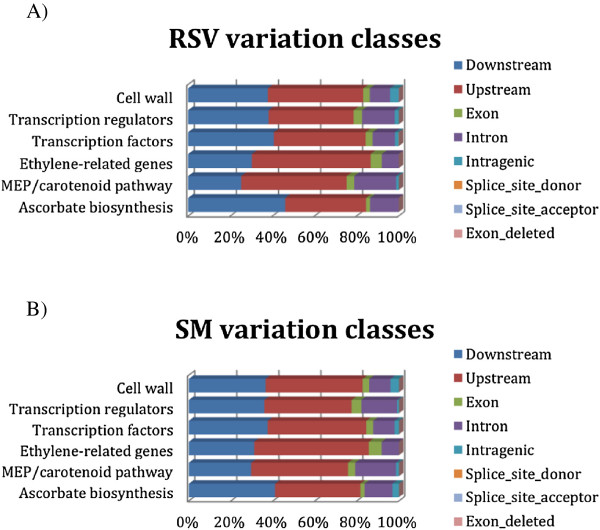
**Classification of variants in fruit quality and ripening related genes.** Classifications of variants were based on the their gene location **A)** Vesuviano (RSV) sequence variants **B)** San Marzano (SM) sequence variants.

**Figure 5:**
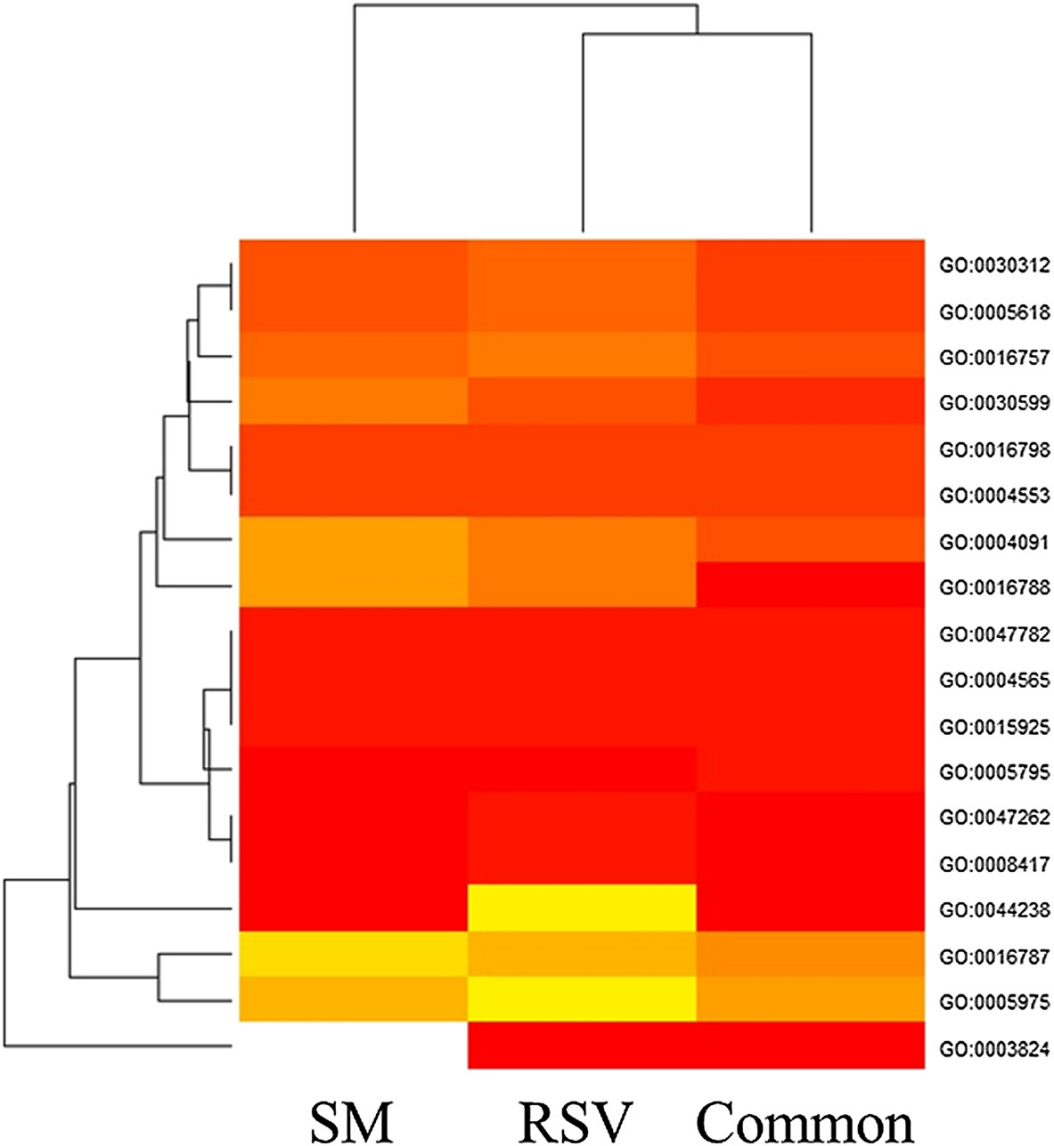
**GO terms enrichment heat map.** Significant GO term classes enriched Vesuviano (RSV) and San Marzano (SM) non-synonymous coding variants are reported Variants found in both varieties respect reference genome are indicated as common; variants found in each variety (RSV or SM) are indicated as specific.

The analysis of genetic differences between RSV and SM genomes and the reference tomato genome SL2.40 has been focused on four gene classes related to fruit quality (ascorbate biosynthesis; MEP/carotenoid pathway; ethylene-related genes; cell wall related genes); transcription factors and transcription regulators potentially involved in fruit ripening process were also included (Table 6). A high percentage of genes belonging to all investigated classes showed variants. On average 10 variations for gene have been identified, ranging from 7 to 14 in RSV and from 7 to 19 in SM. The total number of varied genes is not indicative of specificity of variants for RSV or SM genes. Table 7 indicated that RVS and SM have 2,566 common genes with variants; nevertheless specific varied genes for each variety were also highlighted. Indeed, RSV showed a higher percentage of specific polymorphism (5.6%) compared to SM (2.9%). Interestingly, three ACS genes and a ETR1 gene involved in ethylene biosynthesis varied only in RSV (Additional file 2: Table S6). A high percentage of variations is included in upstream and downstream regions, with values (on total variants belonging to each class) ranging between 23.60% and 61.80% for RSV tomato and from 23.75% and 61.25% for SM tomato (Figure 4). Putative impact of variants has been evaluated, focusing on non-synonymous variants localized in the coding sequence (CDS). The number of genes with non-synonymous variants in the CDS found specifically in RSV or in SM and the number of common variants are reported in Additional file 2: Table S7. In order to understand if an amino acid substitution has an impact on the biological protein function these gene sub-sets have been analyzed using the PROVEAN predictor. Out a total of 386 genes analysed belonging to the selected groups, 45 showed predicted deleterious non-synonymous variations (11.6%) in the coding sequence when translated as amino acid substitutions (Table 8). The transcription factor class showed the highest number of deleterious substitution with 5, 6, and 11 genes in SM, RSV, and both genotypes, respectively. Similarly, deleterious variations for the protein function were observed in genes belonging to the cell wall and transcription regulators categories. Moreover, to highlight if there were gene functional categories susceptible of significant variation among the two tomato varieties and the reference, a singular enrichment analysis (SEA) was performed. The analyses showed that the gene class of transcription factors with non-synonymous variants common to both tomato varieties was enriched for three molecular function GO terms: interleukin-6 receptor binding (GO:0005138), cytokine activity (GO:0005125) and RNA polymerase II transcription elongation factor activity (GO:0016944). RSV transcription regulation variants showed enrichment in molecular GO function for ethylene-binding class (GO:0051740), due to the presence of the gene encoding for the ethylene receptor (Solyc07g056580 histidine kinase-related protein, a variant absent in SM). RSV transcription regulation non-synonymous variants showed enrichment in tight junction class (GO:0005923) because of the presence of the SNF2 helicase gene (Solyc03g095680, histone linker, a variant absent in SM). Figure 5 reports common and variety-specific non-synonymous cell wall coding variants enriched classes. Common non-synonynous variants showed enrichment in molecular function GO terms corresponding to hydrolase activity, hydrolyzing O-glycosyl compounds (GO:0004553), galactosidase activity (GO:0015925), coniferin beta-glucosidase activity (GO:0047782), beta-galactosidase activity (GO:0004565). RSV-specific non-synonymous variants were enriched in fucosyltransferase activity (GO:0008417) and polygalacturonate 4-alpha-galacturonosyltransferase activity (GO:0047262). Indeed, the presence of non-synonymous variants in the fucosyltransferase 7 gene (Solyc03g115830) and in the glycosyltransferase (Solyc07g055930) determined a private significant enrichment in those functional classes.

**Table 6:** Number and percentage of polymorphic genes and number of variants identified for each fruit quality-related class of genes in Vesuviano (RSV) and San Marzano (SM) varieties Data are referred to the tomato reference genome (SL240)

		**RSV**			**SM**		
**Gene class**	**Genes no**	**Polymorphic genes**		**Variants**	**Polymorphic genes**		**Variants**
		**No**	**%**	**No**	**No**	**%**	**No**
Ascorbate biosynthesis	23	18	783	265	18	783	349
MEP/carotenoid pathway	46	35	761	337	34	739	351
Ethylene-related genes	52	48	923	466	44	846	448
Cell wall	718	589	820	5,303	562	783	4,471
Transcription factors	2,025	1,710	844	17,215	1,662	821	15,412
Transcription regulators	434	353	813	2,660	344	793	2,496
**Total**	**3,298**	**2,753**		**26,246**	**2,664**		**23,527**

**Table 7:** Common and specific genes related to fruit quality with variants in Vesuviano (RSV) and San Marzano (SM) varieties

**Gene class**	**Common**	**RSV**	**SM**
	**No**	**No**	**No**
Ascorbate biosynthesis	16	2	2
MEP/carotenoid pathway	33	2	1
Ethylene-related genes	44	4	0
Cell wall	1,600	110	62
Transcription factors	328	25	16
Transcription regulators	545	44	17
**Total**	**2,566**	**187**	**98**

**Table 8:** Fruit quality related genes affected by deleterious mutation in SM, RSV or both varieties

**SM**			
**Gene**	**Annotation**	**Variant**	**Score value**
	**Transcription factors**			
Solyc01g1062302	B3 domain-containing protein	V79F	−3233	
Solyc03g1182902	Auxin response factor 2	S220G	−3818	
Solyc04g0094402	NAC domain protein	Y212C	−3401	
Solyc04g0647701	Zinc finger CCCH domain-containing protein 38	K687Q	−29	
Solyc10g0804101	BZIP transcription factor	T403I	−4147	
**Transcription regulators**				
Solyc07g0519801	Chromodomain-helicase-DNA-binding protein 1-like	A618T	−2833	
Solyc09g0760102	PHD finger family protein	Y390C/L988F	−3167/−6472	
**Cell wall**				
Solyc02g0782301	Glucan synthase like 1	V1659G	−5286	
Solyc05g0518702	Pollen allergen Phl p 11	A134T	−3344	
Solyc05g0554901	Laccase-22	R472H	−49	
**RSV**				
**Gene**	**Annotation**	**Variant**	**Score value**	
**Transcription factors**				
Solyc01g1038301	Zinc finger-homeodomain protein 2	R56Q	−4000	
Solyc03g0971202	Heat stress transcription factor A3	W469L	−4,548	
Solyc07g0550001	Myb-related transcription factor	T110I	−5433	
Solyc08g0052902	BZIP transcription factor 3	E357G	−5548	
Solyc08g0062401	B3 domain-containing protein	R299W	−4223	
Solyc09g0080402	RNA polymerase sigma-70 factor	G436R	−5900	
**Transcription regulators**				
Solyc01g0497401	DNA repair and recombination protein RAD54-like	R155T	−4767	
Solyc02g0646902	Acetyltransferase-like protein	I12T	−3386	
**Cell wall**				
Solyc01g0087201	Mannan endo-1 4-beta-mannosidase	G215S	−5733	
Solyc03g1153101	Expansin	E146G	−3595	
Solyc11g0565901	Cellulose synthase	D100N	−4472	
**Common**				
**Gene**	**Annotation**	**Variant**	**Score value**	
**Transcription factors**				
Solyc00g1983601	Zinc finger CCCH domain-containing protein 30	K188T	−4865	
Solyc02g0773901	WUSCHEL-related homeobox 11	T291K	−2933	
Solyc02g0815202	Nibrin	F236L	−5433	
Solyc03g0439102	BSD domain containing protein	G54C	−8114	
Solyc05g0502202	G-box binding factor 3	N335S	−4567	
Solyc07g0418501	Homeodomain-containing transcription factor FWA	I545T	−4584	
Solyc09g0075502	Zinc finger family protein	V111L	−3000	
Solyc09g0075701	MYB transcription factor	K112I	−6351	
Solyc10g0181101	MADS box transcription factor	L104P	−3739	
Solyc12g0107601	Transcription factor (Fragment)	A36V/G44C/L38P	−2844/−7506/−6344	
Solyc12g0198201	MYB transcription factor	K14R	−2942	
**Transcription regulators**				
Solyc04g0086102	Histone acetyltransferase	P1416L	−7522	
Solyc06g0842502	Kelch repeat and BTB domain-containing	A324T	−2700	
Solyc08g0687701	N-acetyltransferase	W198L	−12149	
Solyc10g0062202	Cell differentiation protein rcd1	Q215E	−2957	
**Ripening ethylene related**				
Solyc01g0791802	Pectinesterase	T334I	−2612	
Solyc01g0811801	Beta-glucosidase	H83Y	−4364	
Solyc01g0915302	Fasciclin-like arabinogalactan protein 13	S201Y	−3582	
Solyc02g0676501	Polygalacturonase 1	G320S	−5337	
Solyc05g0500102	1-aminocyclopropane-1-carboxylate synthase	T82A	−3776	
Solyc05g0525301	Endoglucanase 1	A183V/I97T	−3085/−4045	
Solyc08g0653202	Transmembrane protein 222	V157L	−2583	
Solyc09g0076501	Fasciclin-like arabinogalactan protein 7	S104F	−5217	
Solyc09g0102102	Endoglucanase 1	A306V	−3709	

Data are referred to non-synonymous amino acid variants in CDS regions PROVEAN score threshold = −25 (Variants with score equal or below −25 are considered “deleterious”).

## Discussion

In this work the tomato RVS and SM genomes have been sequenced and assembled using a strategy based on iterative mapping and de novo assembly [14]. This method showed to be less demanding in terms of sequencing depth and multiple libraries construction compared to a complete *de novo* assembly. The catalogue of tomato genetic variants produced using this valuable approach allowed enlarging the list available (http://solgenomics.net/search/markers) with a relative low investment. The magnitude of the number of variants found is not comparable with earlier catalogue, based on transcriptome sequencing or oligonucleotide arrays [28-32]. In addition, other types of variations in CDS sequences were evidenced.

The chromosome pseudomolecules obtained allowed studying with high accuracy genome colinearity useful for gene mapping and marker-assisted breeding. At 40x sequence coverage, we estimated that approximately 99% of the tomato reference genome could be genotyped. Our analysis produced approximately 200,000 SNPs and more than 130,000 indels. In accordance with the high level of homozygosis reported for tomato cultivars [29], a small fraction (approximately 3%) of heterozygous variants or sequence misalignments were identified in either cultivar. Variation in the level of polymorphism among chromosomes was found. Indeed, the chromosome variation could reflect selection history rather than polymorphism discovery [30]. More than 3,000 genes in both varieties showed different similarity values at exon level when compared with reference genome. A slight higher colinearity between SM and the reference genome was found, suggesting a their closer relationship. Genome-wide structural and gene content variations are hypothesized to drive important phenotypic variation within a species [33]. However, in most cases deletions are common to both varieties and their frequency is consistent with previous data on indel errors in the reference genome, and thus we suspect that a percentage of *de novo* assembled sequences corresponds to sequences missing from the reference genome.

Based on the tomato gene model set, a limited number of altered genes were detected in each variety, while 1,934 RSV and 1,707 SM transferred annotations were affected by mutations potentially causing amino acidic substitutions of unknown effect on the protein function. A subset of these SNPs was restricted to a single variety. The study of distribution of variants across the genomes of the sequenced variety is important. Number, location and predicted effects can gain insights in plant diversification. Indeed, the selective forces acting over time on diverse traits could have driven the fixation of positive mutations in each variety. Whether a polymorphic equilibrium is reached depends on the intensity of selection and the relative distances to the *optimum* of the homozygosis at each locus [34].

Analysis of genetic variants for quality related genes showed that genes were differentially affected by genetic variants depending on the functional class they belong to, suggesting different degrees of selection for genetic variants underlying biological processes. We also showed that the position of sequence variants influence the functionality of the encoded protein. Functional variants contributing to deletion in 3′UTR and exon, intron_conserved and exon, intron_conserved region were highlighted; by contrast, a limited number of other intronic/esonic variants were identified. SNPs within the gene classes assessed reflect the fruit quality genetic diversity between RSV and SM varieties. High percent of variation and deleterious substitutions has been found in genes belonging to the transcription factor and transcription regulator classes, such as acetyltrasferase, chromodomain helicase and histone linkers. Interestingly, enrichment for a chromatin remodeler like protein ligase SNF2 in RSV genotypes points out the possibility that the phenotypic differences among these three tomato genotype are mainly due to complex mechanisms of gene regulation and cross-talk. Recently it has been showed that important epigenome modifications are associated with ripening process [35]. The ethylene-related gene class also showed a high number of variants and deleterious substitutions, probably due to the large difference in the ripening process of the two tomato varieties with respect to the reference tomato. In particular, an ACS gene showed a deleterious substitution (T82A) in both varieties and three ACS key genes involved in ethylene biosynthesis varied only in RSV. This is a long-storage tomato variety with extended shelf life. Since ethylene control fruit ripening process [36], polymorphisms detected in these RSV genes should be further explored to understand their involvement in delaying ripening process. Ethylene production is regulated by combinatorial interplay of the ACS polypeptides. Understand how the “ACS symphony orchestra” is coordinated will be a big challenge for the future [37]. Finally, the result of SEA analyse indicated a significant enrichment of the cell wall genes. GO terms corresponding to hydrolase, galactosidase, beta-glucosidase and beta-galactosidase, involved in chemicals breakdown activity inside the fruits, showed significant differences in both varieties. In particular, RSV-specific non-synonymous variants were enriched in genes involved in xyloglucan biosynthesis and homogalacturonan biosynthesis. Genes related to fruit texture has been frequent targets for genetic engineering, with the goal of extending shelf life [38]. Future investigations on these genes and ethylene related genes should be achieved. The regulation of texture and shelf life is complex and performing a deeper analysis of variants discovered could allow a better understanding of the relationship between changes in the textural and shelf life extension [36].

## Conclusions

The genome sequences reported here and the variants catalogue obtained will be useful to identify the molecular basis of gene complex patterns. Further analysis and functional studies will serve as a basis for understanding trait differences, which will facilitate the identification of markers for genomic marker–assisted breeding. Data produced can be also useful to prioritize mutations to reveal a phenotype. Indeed, large-scale TILLING projects can be used to identify gene of interest saturated with mutations [39]. Collectively, sequence and fine annotation analysis performed can be useful to examine the path from sequence variant to phenotype for improving the utility of the tomato as a model for fruit quality. In addition, the genes we identified that are related to ripening and texture characteristics could be used as target for tomato breeding. The local genomes genotyping is also useful for understanding the genomic features that distinguish modern from traditional varieties. Variants specific for SM and RSV might be explored through a high throughput target re-sequencing approach in other varieties in order to verify that they could represent variants characteristics for these two different tomato typologies.

## Competing interests

The authors declare that they have no competing interests.

## Authors’ contributions

EMR was involved in conception and design of study, in interpretation of data and in manuscript writing, SA in gene variants analysis, interpretation of data and in manuscript drafting, FF in acquisition of data and analysis, DR in gene variants analysis and writing, TP in gene annotation process and in writing, TA in CDS mapping analysis and writing, BA in gene variants data interpretation and in manuscript drafting, ZE in sequencing and analysis of data, CML analysis, interpretation of data and in manuscript writing, BG in genome assembling and analysis, DM in experiments design and in critically revision of manuscript; FL conceived the study and coordinated the project and has given final approval of the version to be published. All authors read and approved the final manuscript.

## Supplementary Material

Additional file 1: Figure S1 Fruit morphological characteristics. Click here for file

Additional file 2: Table S1 Sequencing statistics. Statistics of sequencing of Vesuviano (RSV) and San Marzano (SM) cultivars of *Solanum lycopersicon*. **Table S2.** RepeatMasker results statistics. SL2.40 genome is annotated with similar number of mobile elements (63%) of and with similar outstanding proportion of LTR. **Table S3.** Genome reconstruction statistics. Size of the reconstructed chromosomes in San Marzano (SM) and Vesuviano (RSV) respect to Heinz SL2.40 (H) Genome. **Table S4.** Polymorphic regions. General statistics of polymorphic regions detected in RSV and SM genotypes. **Table S5.** Gene Annotation Transfer. The gene annotation was transferred from the reference to the reconstructed cultivar genome on the basis of the putative effect of called mutations. If the mutation had a putative disrupting effect on the CDS the gene was not transferred to the new annotation. **Table S6.** List of varied genes in fruit quality and ripening-related classes. **Table S7.** Non-synonymous exon variants in fruit quality and ripening-related gene classes. Click here for file

Additional file 3: Figure S2 Assembly accuracy. Assembly accuracy (y axis; number of base substitution errors) at 12 stages of the iterative mapping against the reference genome. Click here for file

Additional file 4: Figure S3 Distribution of San Marzano (SM) chromosome 9 SNPs density. Click here for file

## References

[B1] Bai Y Lindhout P Domestication and breeding of tomatoes: what have we gained and what can we gain in the future? Ann Bot-London 2007 100 1085 1094 10.1093/aob/mcm150 PMC2759208 17717024

[B2] Grandillo S Chetelat R Knapp SK Spooner DM Peralta I Cammareri M Perez O Termolino P Tripode P Chiusano ML Ercolano MR Fruschiante L Monte L Pignone D Kole C Solanum section Lycopersicon Wild crop relatives: Genomic and breeding resources, Volume 5 2011 Berlin, Heidelberg: Springer-Verlag 129 216

[B3] Zago F Buone varietà di pomodoro L'Italia Agricola 1912 44 110 456

[B4] Zago F Varietà per la preparazione dei pelati L'Italia Agricola 1929 66 360 362

[B5] Ercolano MR Carli P Soria A Cascone A Fogliano V Frusciante L Barone A Biochemical sensorial and genomic profiling of Italian tomato traditional varieties Euphytica 2008 164 571 582 10.1007/s10681-008-9768-4

[B6] Carli P Barone A Fogliano V Frusciante L Ercolano MR Dissection of genetic and environmental factors involved in tomato organoleptic quality BMC Plant Biol 2011 11 58 10.1186/1471-2229-11-58 21453463 PMC3080294

[B7] Garcia-Martinez S Corrado G Ruiz-Martinez JJ Rao R Diversity and structure of a sample of traditional Italian and Spanish tomato accessions Genet Resour Crop Evol 2013 60 789 798 10.1007/s10722-012-9876-9

[B8] The Tomato Genome Consortium The tomato genome sequence provides insights into fleshy fruit evolution Nature 2012 485 635 641 10.1038/nature11119 22660326 PMC3378239

[B9] Todd PM Alba R The tomato genome fleshed out Nat Biotechnol 2012 30 765 767 10.1038/nbt.2319 22871715

[B10] The 1000 Genomes Project Consortium An integrated map of genetic variation from 1092 human genomes Nature 2012 491 56 65 10.1038/nature11632 23128226 PMC3498066

[B11] Cao J Schneeberger K Ossowski S Günther T Bender S Fitz1 J Koenig D Lanz C Stegle O Lippert C Wang X Ott F Müller J Alonso-Blanco C Borgwardt K Schmid KJ Weigel D Whole-genome sequencing of multiple Arabidopsis thaliana populations Nat Genet 2011 43 956 963 10.1038/ng.911 21874002

[B12] Treangen TJ Salzberg SL Repetitive DNA and next-generation sequencing: computational challenges and solutions Nat Rev Genet 2011 13 36 46 22124482 10.1038/nrg3117 PMC3324860

[B13] Schneeberger K Ossowski S Ott F Klein JD Wang X Lanz C Smith LM Cao J Fitz J Warthmann N Henz SR Huson DH Weigel D Reference-guided assembly of four diverse Arabidopsis thaliana genomes Proc Natl Acad Sci USA 2011 108 10249 10254 10.1073/pnas.1107739108 21646520 PMC3121819

[B14] Gan X Stegle O Behr J Steffen JG Drewe P Hildebrand KL Lyngsoe R Schultheiss SJ Osborne EJ Sreedharan VT Kahles A Bohnert R Jean G Derwent P Kersey P Belfield EJ Harberd NP Kemen E Toomajian C Kover PX Clark RM Rätsch G Mott R Multiple reference genomes and transcriptomes for Arabidopsis thaliana Nature 2011 477 419 423 10.1038/nature10414 21874022 PMC4856438

[B15] Bevan M Genomics: endless variation most beautiful Nature 2011 477 415 416 10.1038/477415a 21938062

[B16] Jiménez-Gómez JM Maloof JN Sequence diversity in three tomato species: SNPs, markers, and molecular evolution BMC Plant Biol 2009 9 85 10.1186/1471-2229-9-85 19575805 PMC3224693

[B17] Lunter G Goodson M Stampy: a statistical algorithm for sensitive and fast mapping of Illumina sequence reads Genome Res 2011 21 936 939 10.1101/gr.111120.110 20980556 PMC3106326

[B18] Gremme G Brendel V Sparks ME Kurtz S Engineering a software tool for gene structure prediction in higher organisms Inf Softw Technol 2005 47 965 978 10.1016/j.infsof.2005.09.005

[B19] Krzywinski M Schein J Birol I Connors J Gascoyne R Horsman D Jones S Marra M Circos: an information aesthetic for comparative genomics Genome Res 2009 19 1639 1645 10.1101/gr.092759.109 19541911 PMC2752132

[B20] Cingolani P Platts A Wang LL Coon M Nguyen T Wang L Land SJ Lu X Ruden DM A program for annotating and predicting the effects of single nucleotide polymorphisms, SnpEff: SNPs in the genome of_*Drosophila melanogaster* strain w1118; iso-2; iso-3 Fly 2012 6 80 92 10.4161/fly.19695 22728672 PMC3679285

[B21] Choi Y Sims GE Murphy S Miller JR Chan AP Predicting the functional effect of amino acid substitutions and indels PLOSOne 2012 7 e46688 10.1371/journal.pone.0046688 PMC3466303 23056405

[B22] Li H Handsaker B Wysoker A Fennell T Ruan J Homer N Marth G Abecasis G Durbin R and 1000 Genome Project Data Processing Subgroup The Sequence alignment/map (SAM) format and SAMtools Bioinformatics 2009 25 2078 2079 10.1093/bioinformatics/btp352 19505943 PMC2723002

[B23] Quinlan AR Hall IM BEDTools: a flexible suite of utilities for comparing genomic features Bioinformatics 2010 26 6 841 842 10.1093/bioinformatics/btq033 20110278 PMC2832824

[B24] Ashburner M Ball CA Blake JA Botstein D Butler H Cherry JM Davis AP Dolinski K Dwight SS Eppig JT Harris MA Hill DP Issel-Tarver L Kasarskis A Lewis S Matese JC Richardson JE Ringwald M Rubin GM Sherlock G Gene ontology: tool for the unification of biology The Gene Ontology Consortium Nat Genet 2000 25 25 29 10.1038/75556 10802651 PMC3037419

[B25] Conesa A Götz S García-Gómez JM Teroll J Talónl M Roblesl M Blast2GO: a universal tool for annotation visualization and analysis in functional genomics research Bioinformatics 2005 21 3674 3676 10.1093/bioinformatics/bti610 16081474

[B26] Du Z Zhou X Ling Y Zhang Z Su Z agriGO: a GO analysis toolkit for the agricultural community Nucl Acids Res 2012 38 W64 W70 20435677 10.1093/nar/gkq310 PMC2896167

[B27] Tipney H Hunter L An introduction to effective use of enrichment analysis software Hum Genomics 2010 4 202 206 10.1186/1479-7364-4-3-202 20368141 PMC3525973

[B28] Hamilton JP Sim SC Stoffel K Single nucleotide polymorphism discovery in cultivated tomato via sequencing by synthesis The Plant Genome 2012 5 17 29 10.3835/plantgenome2011.12.0033

[B29] Sim SC Robbins MD Van Deynze A Michel AP Francis DM Population structure and genetic differentiation associated with breeding history and selection in tomato (Solanum lycopersicum L) Heredity 2011 106 927 935 10.1038/hdy.2010.139 21081965 PMC3186243

[B30] Sim SC Van Deynze A Stoffel K Douches DS Zarka D Ganal MW Chetelat RT Hutton SF Scott JW Gardner RG Panthee DR Mutschler M Myers JR Francis DM High-density SNP genotyping of tomato (Solanum lycopersicum L) reveals patterns of genetic variation due to breeding PLOSOne 2012 7 e45520 10.1371/journal.pone.0045520 PMC3447764 23029069

[B31] Blanca J Cañizares J Cordero L Pascual L Diez MJ Nuez F Variation revealed by SNP genotyping and morphology provides insight into the origin of the tomato PLOSOne 2012 7 e48198 10.1371/journal.pone.0048198 PMC3485194 23118951

[B32] Hirakawa H Shirasawa K Ohyama A Fukuoka H Aoki K Rothan C Sato S Isobe S Tabata S Genome-wide SNP genotyping to infer the effects on Gene functions in tomato DNA Res 2013 20 3 221 233 doi:10.1093/dnares/dst005 10.1093/dnares/dst005 23482505 PMC3686429

[B33] McHale LK Haun WJ Xu WW Bhaskar PB Anderson JE Hyten DL Gerhardt DJ Jeddeloh JA Stupar RM Structural variants in the soybean genome localize to clusters of biotic stress-response genes Plant Physiol 2012 159 1295 1308 10.1104/pp.112.194605 22696021 PMC3425179

[B34] Pavlidis P Metzler D Stephan W Selective sweeps in multilocus models of quantitative traits Genetics 2012 192 225 239 10.1534/genetics.112.142547 22714406 PMC3430538

[B35] Zhong S Fei Z Chen YR Zheng Y Huang M Vrebalov J McQuinn R Gapper N Liu B Xiang J Shao Y Giovannoni JJ Single-base resolution methylomes of tomato fruit development reveal epigenome modifications associated with ripening Nat Biotechnol 2013 31 154 159 10.1038/nbt.2462 23354102

[B36] Klee HJ Giovannoni JJ Genetics and control of tomato fruit ripening and quality attributes Annu Rev Genet 2011 45 41 59 10.1146/annurev-genet-110410-132507 22060040

[B37] Tsuchisaka A Yu G Jin H Alonso JM Ecker JR Zhang X Gao S Theologis A A combinatorial interplay among the 1-aminocyclopropane-1-carboxylate isoforms regulates ethylene biosynthesis in Arabidopsis thaliana Genetics 2009 183 979 1003 10.1534/genetics.109.107102 19752216 PMC2778992

[B38] Matas A Gapper N Chung M Giovannoni J Rose JKC Biology and genetic engineering of fruit maturation for enhanced quality and shelf-life Curr Opin Biotech 2009 20 197 203 10.1016/j.copbio.2009.02.015 19339169

[B39] Ng PC Henikoff S Predicting the effects of amino acid substitutions on protein function Annu Rev Genomics Hum Genet 2006 7 61 80 10.1146/annurev.genom.7.080505.115630 16824020

