# Supplementary material for: Patchwork sequencing of tomato San Marzano and Vesuviano varieties highlights genome-wide variations
Source: BMC Genomics. 2014 Feb 18;15:138. doi: 10.1186/1471-2164-15-138 (PMC3936818; doi:10.1186/1471-2164-15-138)
Supplement: Additional file 1: Figure S1 — Fruit morphological characteristics. [file 1471-2164-15-138-S1.pptx]

## Slide 1
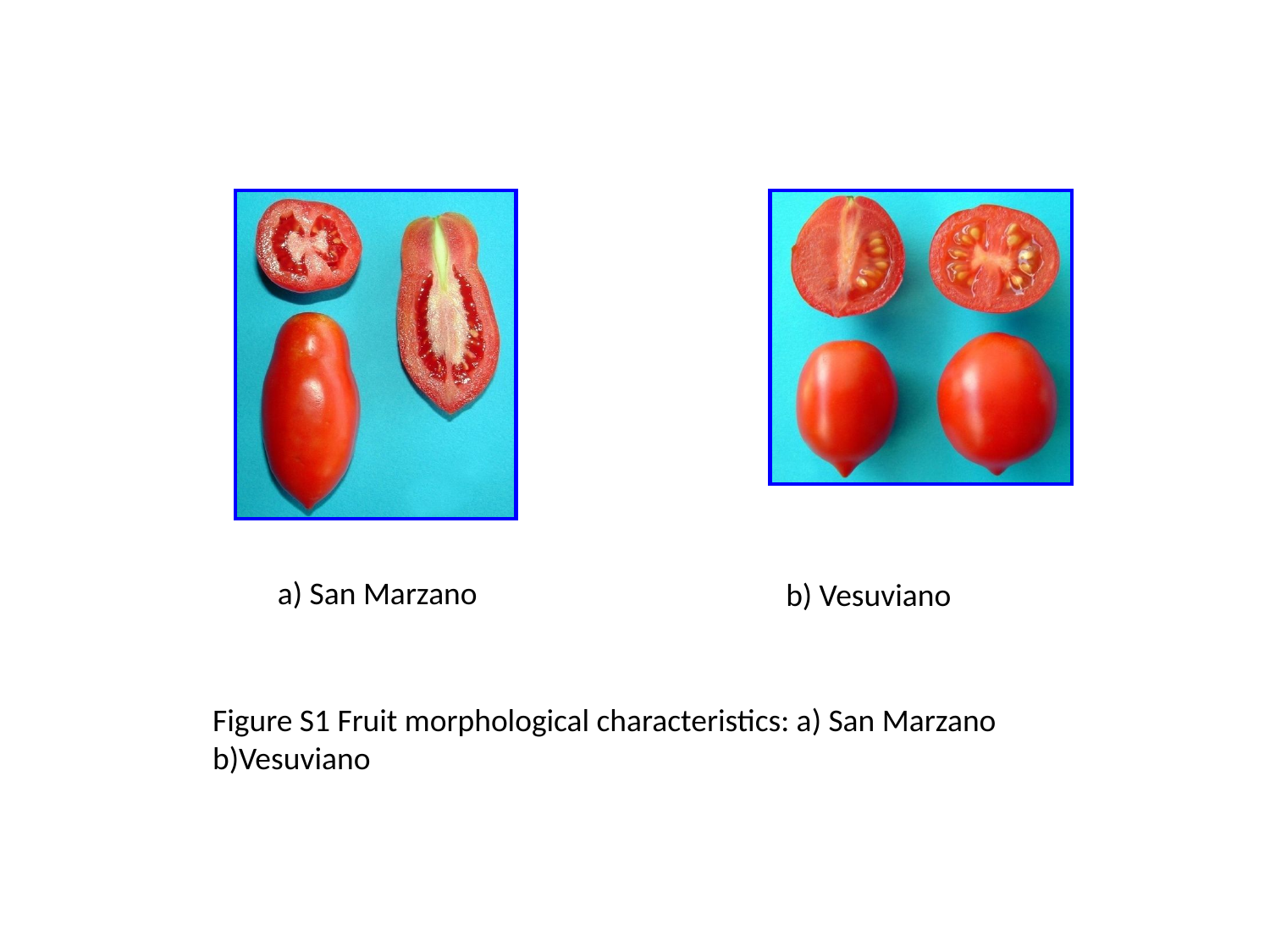

a) San Marzano
b) Vesuviano
Figure S1 Fruit morphological characteristics: a) San Marzano b)Vesuviano
