# Supplementary material for: Patchwork sequencing of tomato San Marzano and Vesuviano varieties highlights genome-wide variations
Source: BMC Genomics. 2014 Feb 18;15:138. doi: 10.1186/1471-2164-15-138 (PMC3936818; doi:10.1186/1471-2164-15-138)
Supplement: Additional file 3: Figure S2 — Assembly accuracy. Assembly accuracy (y axis; number of base substitution errors) at 12 stages of the iterative mapping against the reference genome. [file 1471-2164-15-138-S3.pptx]

## Slide 1
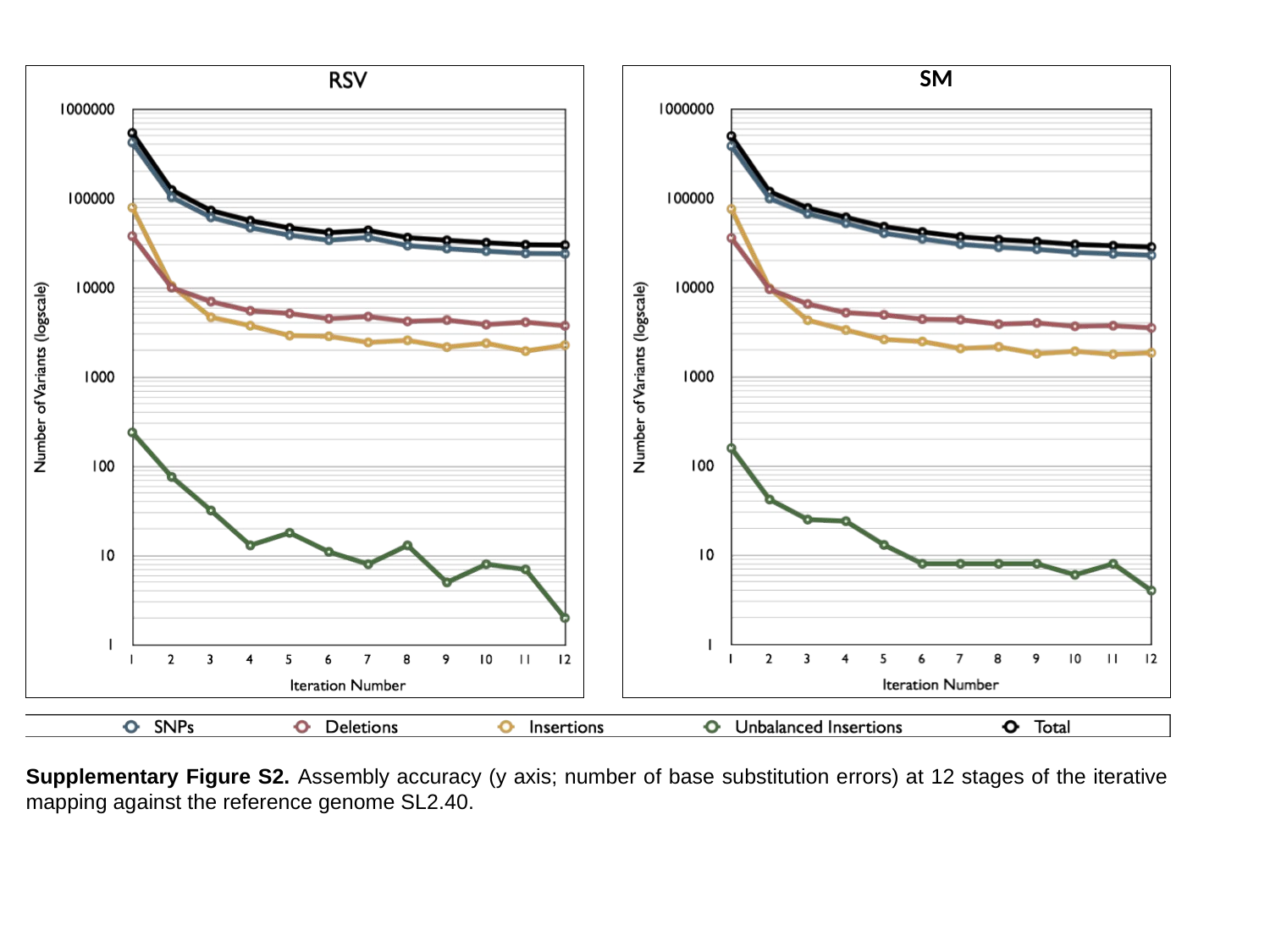

SM
Supplementary Figure S2. Assembly accuracy (y axis; number of base substitution errors) at 12 stages of the iterative mapping against the reference genome SL2.40.
