# Supplementary figures and images for: Patchwork sequencing of tomato San Marzano and Vesuviano varieties highlights genome-wide variations
Source: BMC Genomics. 2014 Feb 18;15:138. doi: 10.1186/1471-2164-15-138 (PMC3936818; doi:10.1186/1471-2164-15-138)

## Slide 1
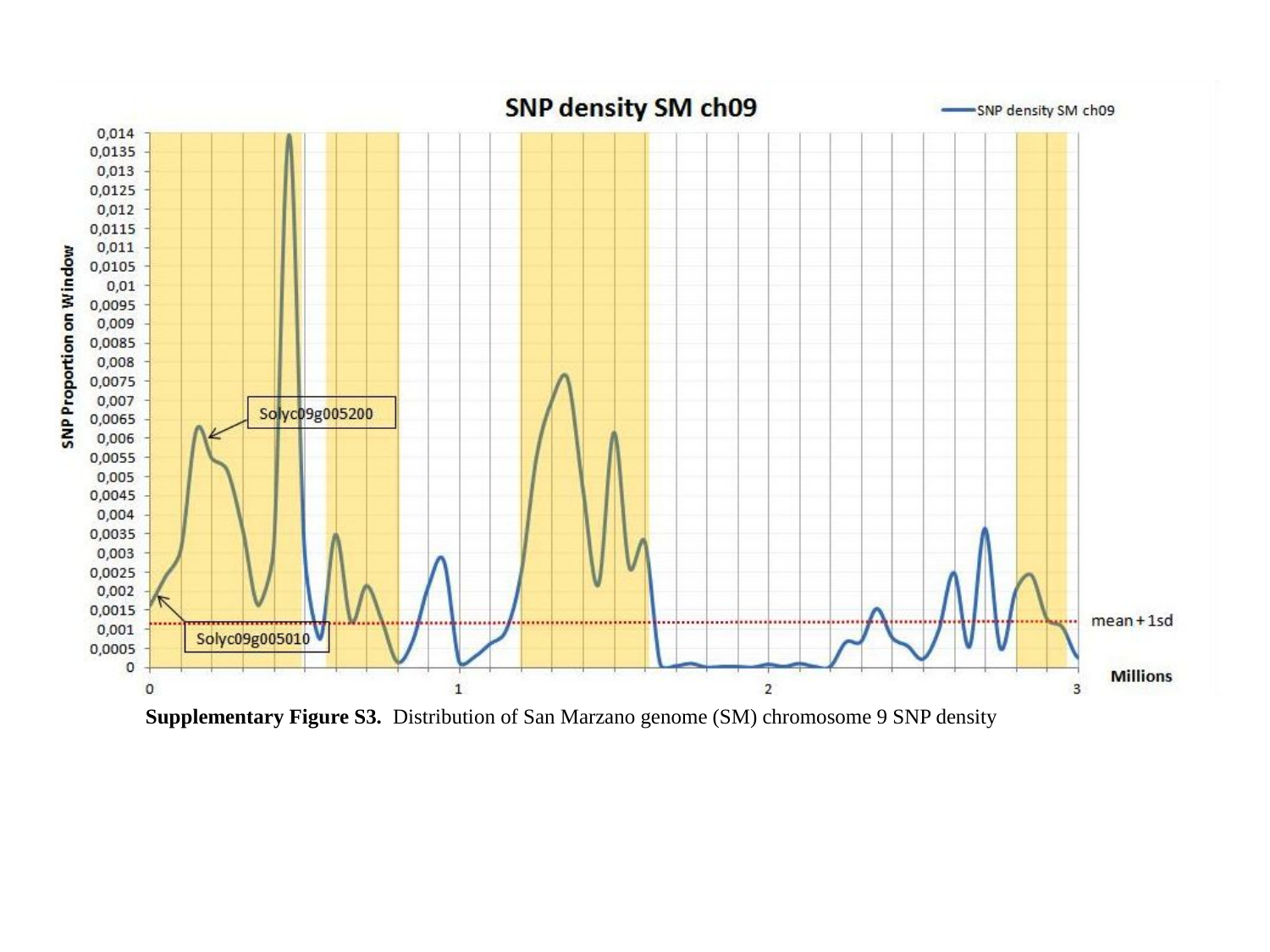

Supplementary Figure S3. Distribution of San Marzano genome (SM) chromosome 9 SNP density

Supplement: Additional file 4: Figure S3 — Distribution of San Marzano (SM) chromosome 9 SNPs density. [file 1471-2164-15-138-S4.pptx]
